# Clinical features and genetic spectrum of a multicenter Chinese cohort with myotonic dystrophy type 1

**DOI:** 10.1186/s13023-024-03114-z

**Published:** 2024-03-07

**Authors:** Huahua Zhong, Li Zeng, Xuefan Yu, Qing Ke, Jihong Dong, Yan Chen, Lijun Luo, Xueli Chang, Junhong Guo, Yiqi Wang, Hui Xiong, Rongrong Liu, Changxia Liu, Jibao Wu, Jie Lin, Jianying Xi, Wenhua Zhu, Song Tan, Fuchen Liu, Jiahong Lu, Chongbo Zhao, Sushan Luo

**Affiliations:** 1 Huashan Rare Disease Center and Department of Neurology, Huashan Hospital, National Center for Neurological Disorders, Fudan University, Shanghai, China; 2 Department of Neurology, Sichuan Provincial People’s Hospital, University of Electronic Science and Technology of China, Sichuan, China; 3 Department of Neurology and Neuroscience Center, The First Affiliated Hospital of Jilin University, Jilin, China; 4 Department of Neurology, The First Affiliated Hospital, Zhejiang University School of Medicine, Zhejiang, China; 5 Department of Neurology, Zhongshan Hospital Fudan University, Shanghai, China; 6 Department of Neurology, Tongji Hospital, Tongji University, Shanghai, China; 7 Department of Neurology, Wuhan No.1 Hospital, Huazhong University of Science and Technology, Hubei, China; 8 Department of Neurology, The First Hospital of Shanxi Medical University, Shanxi, China; 9 Department of Neurology, Zhejiang Provincial People’s Hospital, Hangzhou Medical College, Zhejiang, China; 10 Department of Pediatrics, Peking University First Hospital, Beijing, China; 11 Department of Neurology, Shaoxing Second Hospital, Zhejiang, China; 12 Department of Neurology, Yancheng First People’s Hospital, Jiangsu, China; 13 Department of Neurology, Chenzhou First People’s Hospital, Hunan, China; 14 Department of Neurology, Qilu Hospital, Shandong University, Shangdong, China

## Abstract

**Background:**

As the most common subtype of adult muscular dystrophy worldwide, large cohort reports on myotonic dystrophy type I (DM1) in China are still lacking. This study aims to analyze the genetic and clinical characteristics of Chinese Han DM1 patients.

**Methods:**

Based on the multicenter collaborating effort of the Pan-Yangtze River Delta Alliance for Neuromuscular Disorders, patients with suspected clinical diagnoses of DM1 were genetically confirmed from January 2020 to April 2023. Peak CTG repeats in the DMPK gene were analyzed using triplet repeat-primed PCR (TP-PCR) and flanking PCR. Time-to-event analysis of onset age in females and males was performed. Additionally, detailed clinical features and longitudinal changes from the disease onset in 64 DM1 patients were retrospectively collected and analyzed. The Epworth Sleepiness Scale and Fatigue Severity Scale were used to quantify the severity of daytime sleepiness and fatigue.

**Results:**

Among the 211 genetically confirmed DM1 patients, the mean age at diagnosis was 40.9 ± 12.2 (range: 12–74) with a male-to-female ratio of 124:87. The average size of CTG repeats was 511.3 (range: 92–1945). Among the DM1 patients with comprehensive clinical data (n = 64, mean age 41.0 ± 12.0), the age at onset was significantly earlier in males than in females (4.8 years earlier, *p* = 0.026). Muscle weakness (92.2%), myotonia (85.9%), and fatigue (73.4%) were the most prevalent clinical features. The predominant involved muscles at onset are hands (weakness or myotonia) (52.6%) and legs (walking disability) (42.1%). Of them, 70.3% of patients had daytime sleepiness, 14.1% had cataract surgery, 7.8% used wheelchairs, 4.7% required ventilatory support, and 1.6% required gastric tubes. Regarding the comorbidities, 4.7% of patients had tumors, 17.2% had diabetes, 23.4% had dyspnea, 28.1% had intermittent insomnia, 43.8% experienced dysphagia, and 25% exhibited cognitive impairment. Chinese patients exhibited smaller size of CTG repeats (468 ± 139) than those reported in Italy (613 ± 623), the US (629 ± 386), and Japan (625 [302, 1047]), and milder phenotypes with less multisystem involvement.

**Conclusion:**

The Chinese Han DM1 patients presented milder phenotypes compared to their Caucasian and Japanese counterparts. A male predominance and an early age of onset were identified in male Chinese Han DM1 patients.

## Background

Myotonic dystrophy (DM) is the most prevalent type of muscular dystrophy in adults worldwide (0.37–36.29 cases per 100,000) [1]. It is more than just a progressive degenerative disease confined in skeletal muscles. Multiorgan involvement is highly common in DM patients, encompassing conditions such as cataracts, cardiac conduction abnormalities, infertility, insulin resistance, and so on [2]. DM can be further categorized into DM1 and DM2 according to the different responding genes. DM1 is caused by an expansion of cytosine-thymine-guanine (CTG) trinucleotide repeats in the 3′-untranslated region of the *DMPK* gene and typically presents with more severe symptoms and a higher prevalence than DM2, which is caused by tetranucleotide repeat expansion located in the *CNBP* gene. Normal DMPK gene alleles contain 5–34 CTG repeats, while repeats ranging from 51 to 100 are defined as protomutations. Typically, longer CTG expansions in *DMPK* are responsible for an earlier onset phenotype and severe forms of DM1. These range from late-onset DM1 (100–600 repeats) to adult DM1 (250–750 repeats), childhood DM1 (400–1000 repeats), and congenital DM1 (> 750 repeats) [3]. Most DM1 patients become symptomatic during the second, third, or fourth decade of life, which is termed adult DM1 (classical DM1).

DM1 is much less prevalent in Asia. Compared to the reported global prevalence (9.27 per 100,000), the prevalence of DM1 is 0.77, 0.45, and 0.37 per 100,000 in South Korea, Taiwan, and Hong Kong, respectively [4–6]. Apart from the disease prevalence, clinical features in non-Caucasian DM1 were considerably different from their Caucasian counterparts. For instance, cardiac defects, cataracts, and sleep disturbances were less common in a retrospective study of 37 Chinese DM1 patients [7]. Similarly, a lower prevalence of conduction abnormalities and arrhythmia was reported in a South Korean nationwide study [4]. Hence, the clinical and genetic features of non-Caucasian DM1 patients could expand the global spectrum of DM1. To evaluate the genetic and clinical features of Chinese DM1 patients, we performed this alliance-based multicenter cohort study in genetically confirmed DM1 patients (n = 211). Among them, clinical features of 64 DM1 patients including multi-organ symptoms, age of onset, comorbidities, fatigue and sleepiness scales, and current medications were analyzed.

## Method

Patients with DM1 were enrolled from January 2020 to April 2023 through the collaborative efforts of the Pan-Yangtze River Delta Alliance for Neuromuscular Disorders (PYDAN) network in China [8–10]. Written informed consent to be included in the study was obtained from all patients. The study was approved by the ethics committee of Huashan Hospital (2020–008) and other participating hospitals and was registered on clinicaltrials.gov (NCT06101940). The clinical diagnosis of DM1 was based on classical clinical presentations, electromyography, and family history.

The genetic diagnosis of DM1 was set at an abnormal CTG expansion in the *DMPK* gene (≥ 50 repeats). The peak CTG repeats were measured using flanking PCR and triplet repeat-primed PCR (TP-PCR) [11]. All blood samples were collected and analyzed by the Shanghai Amplicon-gene medical test laboratory using a self-developed DMPK sizing kit. The TP-PCR cycle comprised of an initial denaturation step at 94 °C for 30 s followed by 38 cycles of 94 °C for 30 s, 66 °C for 90 s, with a final extension step at 4 °C for 10 min. TP-PCR amplification products were analyzed by capillary electrophoresis, and the final peak of each sample was identified based on signal height and/or peak morphology [12].

Detailed clinical information of 64 DM1 patients was analyzed, which included: (1) demographics; (2) clinical features (first symptom, myotonia, cognitive impairment, tumor, glucose metabolism, daily sleep, histories of cataract surgery, wheelchair use, cardiac pacemaker implantation, and the use of gastric tubes and respirators); (3) clinical scales including the Epworth Sleepiness Scale (ESS) and Fatigue Severity Scale (FSS) which were used to quantitatively measure daytime sleepiness and fatigue respectively. Daytime sleepiness was defined as an ESS score ≥ 36, and fatigue was defined as an FSS score ≥ 10 based on the suggested threshold scores [13, 14]. Additionally, current medication and main diet were also analyzed. The onset age of DM1 was defined as the onset of noticeable clinical symptoms.

All data and figures were analyzed and generated using R (version 4.3.1). The time-to-event analysis of onset age with respect to sex was conducted using the Log-rank test, utilizing the ‘survminer’ R package. Comparisons of clinical features between true and absent groups, in terms of onset age, were calculated using the Wilcoxon test and the ‘ggpubr’ R package. Additionally, mean values were reported as mean ± SD, and median values were reported as median [0.25 quantile, 0.75 quantile].

## Results

**Fig. 1:**
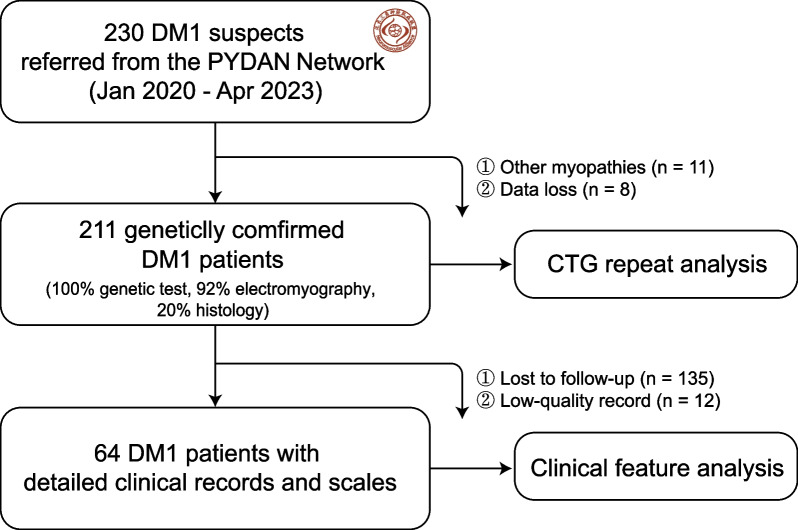
Study workflow

**Fig. 2:**
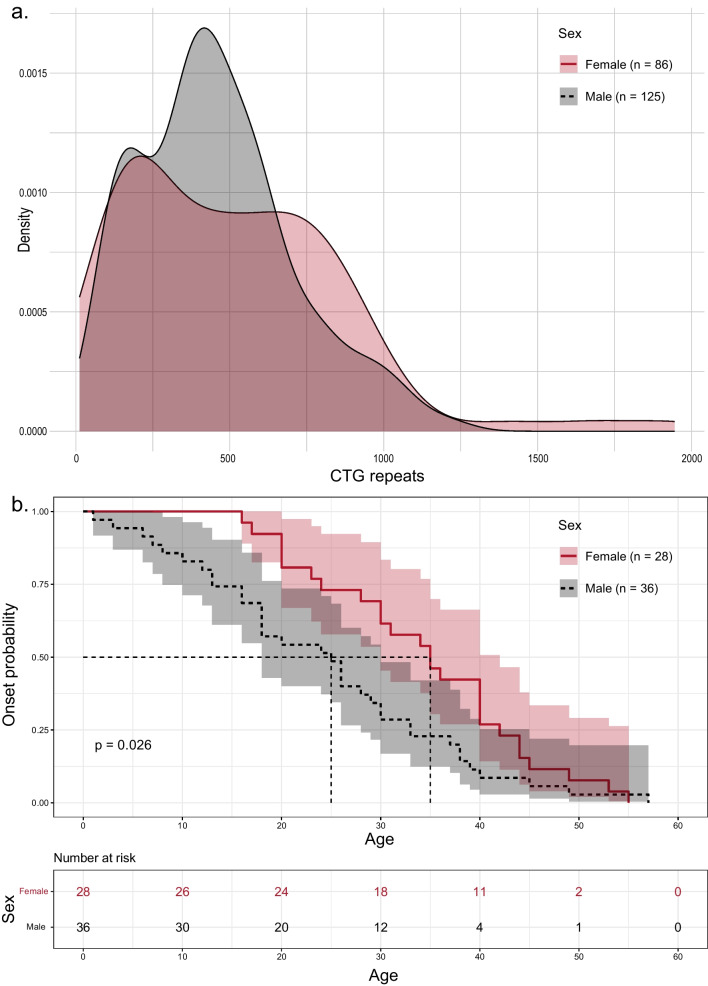
Gender difference in Chinese Han myotonic dystrophy type I (DM1) patients. **a** Density plot of blood peak CTG repeats in male and female DM1 patients. **b** Time-to-event analysis of onset age in female and male DM1 patients. (n = 28 and 36)

Among 230 Chinese Han individuals with clinically suspected DM1, 211 cases were finally genetically confirmed (91.7%). These patients were from 11 provinces across China (except the Northwest region), most of whom (75%) were from East China (Fig. 1). This gave a minimal disease prevalence of 0.13/100,000. The mean age in 211 DM1 patients was 40.9 ± 12.2 and the male-to-female ratio was 124:87. Among them, 92.2% (195/211) had myotonic discharges and myogenic changes in electromyography studies and 20.4% (42/211) had a positive family history. The mean peak CTG repeat length in the DMPK gene was 511.3, ranging from a minimum of 92 to a maximum of 1945 repeats. The distribution of peak CTG repeats was similar between male and female patients, with an average length of approximately 500 repeats (Fig. 2a). More specifically, the average peak CTG repeats were 454.6 ± 248.4 in 124 male patients (mean age 40.9 ± 12.9) and 511.3 ± 358.8 in 87 female patients (mean age 42.0 ± 11.2). However, a time-to-event analysis of onset age by sex revealed an earlier onset in males by 4.8 years (average onset age) (*p* = 0.026) in those 64 patients with detailed clinical information (Fig. 2b).

Among the 64 DM1 patients with detailed clinical data (mean age 41.0 ± 12.0, male: female ratio = 36: 28, CTG repeats 468 ± 139), the most common clinical features observed were muscle weakness (92.2%), myotonia (85.9%), fatigue (73.4%) (Table 1). The onset symptoms mostly presented in hand muscles (weakness or myotonia) (52.6%) and legs (walking disability) (42.1%), and others included vision impairment, persistent fatigue, or cognitive decline. Additionally, 70.3% of patients had daytime sleepiness, 14.1% had undergone cataract surgery, 7.8% used wheelchairs, 4.7% required respirators, and 1.6% needed gastric tubes. Furthermore, 31.3% of patients had frequent heart palpitations (no cardiac device implantation or ablation procedures), 4.7% of patients had tumors (thyroid cancer), 17.2% had diabetes, 23.4% experienced dyspnea, 28.1% occasionally suffered from insomnia, 43.8% reported dysphagia, and 25% exhibited cognitive impairment, primarily in the form of memory decline. In summary, 7.8% of patients were highly disabled as defined by being wheelchair-bound, requiring ventilatory support, or using a gastric tube.

**Table 1:** The distribution of DM1 clinical features based on CTG repeats ranges

Feature	Total patients (n = 64)	CTG repeats 100–300 (n = 18)	CTG repeats 300–600 (n = 27)	CTG repeats 600–1900 (n = 19)	*p*
Age (mean (SD))	41 (12)	44 (15)	39 (11)	43 (9)	0.31
Male (%)	36 (56.2)	11 (61.1)	19 (70.4)	6 (31.6)	0.02
CTG repeat (mean (SD))	468 (139)	174 (44)	447 (81)	754 (105)	0.33
Onset age (%)	29 (13)	38 (11)	25 (12)	31 (13)	0.24
Muscle weakness	59 (92.2)	15 (83.3)	25 (92.6)	19 (100)	0.42
Myotonia (%)	55 (85.9)	16 (88.9)	25 (92.6)	14 (73.7)	0.18
Fatigue (%)	47 (73.4)	11 (61.1)	21 (77.8)	15 (78.9)	0.38
Heart palpitation (%)	20 (31.3)	8 (44.4)	9 (33.3)	3 (15.8)	0.16
Cataract surgery (%)	9 (14.1)	3 (16.7)	4 (14.8)	2 (10.5)	0.86
Tumor (%)	3 (4.7)	1 (5.6)	0 (0.0)	2 (10.5)	0.25
Diabetes (%)	11 (17.2)	5 (27.8)	5 (18.5)	1 (5.3)	0.19
Dyspnea (%)	15 (23.4)	4 (22.2)	6 (22.2)	5 (26.3)	0.94
Insomnia (%)	18 (28.1)	7 (38.9)	7 (25.9)	4 (21.1)	0.46
Daytime sleepiness (%)	45 (70.3)	13 (72.2)	23 (85.2)	9 (47.4)	0.02
FSS score (mean (SD))	42.02 (15.01)	42.44 (14.96)	43.04 (14.29)	44.42 (16.44)	0.62
ESS score (mean (SD))	10.50 (6.18)	11.50 (7.67)	10.85 (5.62)	9.05 (5.40)	0.46

In general, patients with higher CTG repeats were more prone to exhibiting severe symptoms. FSS, Fatigue Severity Scale; ESS, Epworth Sleepiness Scale

**Fig. 3:**
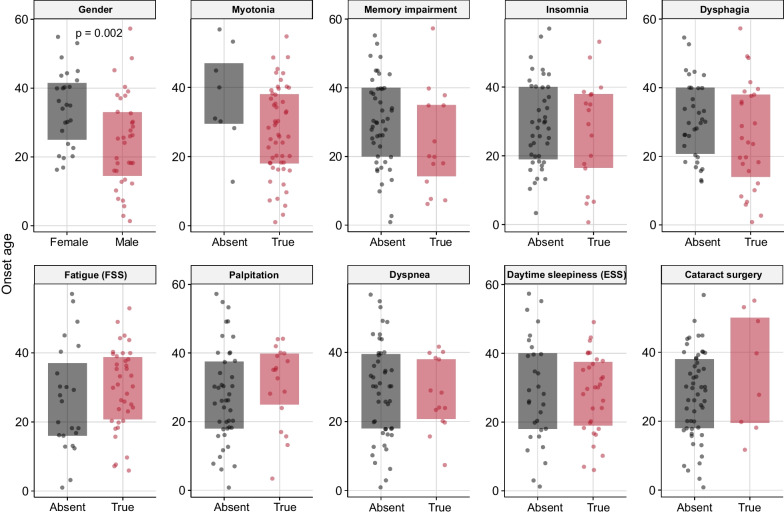
Onset age difference in various clinical features in Chinese Han DM1 patients. Daytime sleepiness was defined as an Epworth Sleepiness Scale (ESS) score ≥ 36, and fatigue was defined as a Fatigue Severity Scale (FSS) score ≥ 10, in accordance with the suggested threshold scores provided by the scales

In terms of medication, 51.6% of patients took mexiletine, and 26.6% took coenzyme Q10. There were no food restrictions for most patients (84.4%). However, 3.1% of patients were meat-oriented, 6.3% of patients were vegetable-oriented, and 6.3% of patients had swallowing difficulty with food restricted in soft fluid. There were no apparent correlations between peak CTG repeats in the DMPK gene and the severity of symptoms. However, patients with an earlier age of onset were prone to present symptoms including myotonia, memory impairment, insomnia, and dysphagia (Fig. 3).

Compared to adult DM1 patients with the same age at diagnosis and onset reported in other ethnicities (Table 2), Chinese DM1 exhibited fewer CTG repeats (468 ± 139) than those reported in Italy (613 ± 623) [15], the US (629 ± 386) [16], and Japan (625 [302, 1047]) [17]. More importantly, the Chinese patients also presented less multisystem impairments, with lower proportions of cataracts (14–30%) and cardiac defects (35%) reported compared to their Caucasian and Japanese counterparts.

**Table 2:** CTG repeats in the DMPK gene and clinical feature comparisons among different ethnicities

References	Region/country	PMID	Sample size	Female%	Age	Age of onset	Blood CTG repeats	Top three most prevalent symptoms	First symptom	Cataract%	Cardiac defects%	Abnormal glucose metabolism%	Daytime sleepiness%	Cognitive impairment%	Cancer%
Groh et al. [16]	US	21484823	406	49%	42 ± 12	26 ± 15	629 ± 386	NA	NA	NA	46%	NA	NA	NA	NA
Peric et al. [24]	Serbia	22695270	120	42%	46 ± 12	NA	NA	NA	NA	78%	68%	NA	NA	61%	NA
Petri et al. [20]	Denmark	24704412	129	50%	44 ± 15	NA	NA	NA	NA	NA	55%	NA	NA	NA	NA
Lu et al. [7]	China	30276279	37	27%	NA	22 [14, 30]	NA	Myotonia, muscle weakness, forehead hair loss	Myotonia	32%	35%	NA	32%	NA	NA
Wood et al. [22]	UK	28397002	556	51%	41 ± 17	26 ± 16	364 ± 266	Fatigue, daytime sleepiness myotonia	NA	26%	48%	NA	78%	NA	NA
Hagerman et al. [25]	North America	30677147	457	60%	45 ± 15	27 ± 15	NA	Muscle weakness, fatigue, daytime sleepiness	NA	NA	NA	14%	87%	63%	NA
Rossi et al. [15]	Italy	30798109	268	44%	46 ± 13	25 ± 13	613 ± 623	NA	NA	NA	31%	NA	63%	NA	NA
Papadimas et al. [26]	Greek	36555146	434	52%	NA	26 ± 15	NA	Muscle Weakness, myotonia hypotonia	Muscle weakness	28%	19%	4%	8%	1%	1%
Yamauchi et al. [17]	Japan	36762492	809	51%	44 [36, 52]	30 [20, 39]	625 [302,1047]	NA	NA	46%	79%	21%	71%	NA	4%
Zhong et al. (This study)	China	NA	64	56%	41 ± 12	29 ± 13	468 ± 139	Muscle weakness, myotonia, fatigue	Muscles weakness	14%	NA	17%	70%	25%	5%

Mean values are reported as mean ± SD, and median values are reported as median [0.25 quantile, 0.75 quantile]

## Discussion

This retrospective cross-sectional study primarily analyzed the genetic and clinical features of Chinese DM1 patients, revealing two key characteristics among our patients. First, there is a relatively less severe form of DM1 in Chinese patients compared to their Caucasian counterparts. Second, there is a male dominance in sex composition and disease severity among our DM1 cohort.

The overall milder form of DM1 in Chinese patients may be attributed to the lower CTG repeats in the DMPK gene. Previous studies have reported significantly lower CTG repeat numbers (less than 19 times) in the Chinese Han normal population (1.0%) when compared to Caucasians (10.0%) and Japanese (8.5%) [18, 19]. This difference in CTG repeat length may also explain the lower estimated prevalence and fewer previous reports of Chinese Han DM1 patients. The lower CTG repeat numbers also influence the most common symptom composition in Chinese Han patients. Lu et al. reported lower rates of cataracts (32.4%), cardiac defects (16.2%), and daytime sleepiness (32.4%) in a cohort of 37 Chinese Han DM1 patients [7]. These three key features are typically common in Caucasians and Japanese DM1, often with a proportion that exceeds 50% [17, 20]. The results of our study are generally consistent with those of Lu et al., although we identified a much higher prevalence of daytime sleepiness (70.3%). This difference might be attributed to our use of a more objective method, the ESS score, to measure daytime sleepiness. Additionally, an increased proportion of muscle weakness and higher FSS scores, along with the increasing CTG repeats were identified in our cohort, which supports the correlation between CTG repeats and severity.

The predominance of males in both sex composition and severity is evident in our DM1 cohort. While gender differences may not be apparent in some populations, a nationwide French study of 1409 adult DM1 patients identified gender as a modifying factor influencing DM1 phenotype severity and mortality. They found that male patients were more likely to exhibit more severe muscular disability with marked myotonia, muscle weakness, cardiac issues, and respiratory involvement compared to female patients [21]. A similar myotonia situation was also reported in a UK Myotonic Dystrophy Patient Registry study (n = 556) [22]. Our study also revealed a higher severity of DM1 in male patients. It’s possible that sex hormones may play a role in modifying the myotonic phenotype by affecting ion channels [23]. However, further clinical, and basic research is needed to fully explain this phenomenon.

This study has several limitations. First, flanking PCR and TP-PCR are not suitable for the accurate analysis of somatic mosaicism in CTG repeats. The estimated progenitor allele length could not be determined in this study. Second, the absence of detailed survey responses in some patients may cause information and selection biases in this study. Since the patients with severe cognitive impairment may not respond to our survey in time, the overall DM1 phenotypes of the Chinese population may be more severe. Fourth, clinical data was retrospectively collected, thus we can not collect enough baseline variables to explore the factors affecting the onset age and other clinical outcomes.

## Conclusions

In summary, this retrospective cohort study summarized the genetic and clinical features of Han Chinese DM1 patients. Chinese DM1 patients exhibited a milder form of the condition compared to their Caucasian and Japanese counterparts and displayed a different clinical phenotype. This complements the global clinical spectrum of DM1.

## Data Availability

For privacy reasons, the data is available from the corresponding author upon request.
